# Compression stress-strain curve of lithium slag recycled fine aggregate concrete

**DOI:** 10.1371/journal.pone.0302176

**Published:** 2024-04-18

**Authors:** Xue-Bin Chen, Jiong-Feng Liang, Wei Li

**Affiliations:** 1 Faculty of Civil & Architecture Engineering, East China University of Technology, Nanchang, P.R. China; 2 College of Civil Engineering and Architecture, Wenzhou University, Wenzhou, P.R. China; 3 Key Laboratory of Engineering and Technology for Soft Soil Foundation and Tideland Reclamation of Zhejiang Province, Wenzhou, P.R. China; University of Malaya, MALAYSIA

## Abstract

As one of the key materials used in the civil engineering industry, concrete has a global annual consumption of approximately 10 billion tons. Cement and fine aggregate are the main raw materials of concrete, and their production causes certain harm to the environment. As one of the countries with the largest production of industrial solid waste, China needs to handle solid waste properly. Researchers have proposed to use them as raw materials for concrete. In this paper, the effects of different lithium slag (LS) contents (0%, 10%, 20%, 40%) and different substitution rates of recycled fine aggregates (RFA) (0%, 10%, 20%, 30%) on the axial compressive strength and stress-strain curve of concrete are discussed. The results show that the axial compressive strength, elastic modulus, and peak strain of concrete can increase first and then decrease when LS is added, and the optimal is reached when the LS content is 20%. With the increase of the substitution rate of RFA, the axial compressive strength and elastic modulus of concrete decrease, but the peak strain increases. The appropriate amount of LS can make up for the mechanical defects caused by the addition of RFA to concrete. Based on the test data, the stress-strain curve relationship of lithium slag recycled fine aggregate concrete is proposed, which has a high degree of agreement compared with the test results, which can provide a reference for practical engineering applications. In this study, LS and RFA are innovatively applied to concrete, which provides a new way for the harmless utilization of solid waste and is of great significance for the control of environmental pollution and resource reuse.

## 1. Introduction

Concrete is the most common building material in the construction industry and the second largest consumer in the world, with around 10 billion tons of concrete produced worldwide every year and showing an upward trend every year [1], posing a huge threat to global warming. As an important cementitious material, cement has been widely used in concrete and mortar. As a major cement producer, China’s cement output in 2022 attained 2.13 billion tons, accounting for about 51.17% of the global output, ranking first in the world [2]. The traditional production of cement is based on the calcination method, which will produce a large amount of CO_2_ in the process, which aggravates the completion of China’s "carbon peak" and "carbon neutrality" goals. Human beings will produce a large amount of industrial waste in production and construction, such as fly ash [3], LS [4], copper tailings [5], etc. In addition, according to statistics, the annual consumption of river sand in the world is about 40 billion to 50 billion tons [6], and river sand alone cannot meet the demand, so there is an urgent need to develop new materials as a substitute for river sand, such as desert sand [7], coconut shells [8], recycled fine aggregates [9], etc. Large amounts of river sand can also pollute water sources and cause problems such as the safety of river embankments. At present, there are a large number of buildings around the world that need to be demolished and waste concrete will be produced in the process. The traditional way of disposing of waste concrete is to landfill or build roads, which not only leads to a large number of waste concrete and is not benignly utilized, but also easily causes problems such as air, soil, and water pollution while occupying land resources. The storage and disposal of this industrial waste, as well as waste concrete, is one of the biggest environmental challenges facing the world today.

In China, with the rapid development of the new energy field, the market demand for lithium batteries is becoming more and more vigorous [10], and a large amount of lithium slag industrial waste will be generated in the process of producing lithium batteries. According to relevant statistics, about 8–10 tons of LS can be produced for every 1 ton of lithium carbonate produced [11]. At present, most of the LS is treated by accumulation, landfill, etc., which will not only lead to a waste of land resources but also cause great harm to the surrounding ecological environment [12, 13]. Since China is the world’s largest producer of lithium carbonate [14, 15], according to China’s industrial development statistics in 2021, the annual output of lithium carbonate will exceed 240,000 tons, mainly distributed in Jiangxi, Xinjiang, and other places [16]; Therefore, it is necessary to find an effective way to utilize LS to promote the sustainable development of the lithium industry.

The mineral composition of LS is mainly composed of oxides such as silicon, aluminum, and calcium [17, 18], which is very similar to the mineral composition of cement. These also makes many researchers use LS as a mineral admixture to partially replace cement, to reduce cement as a necessary binder material in concrete production, thereby reducing construction costs and helping to achieve the goal of carbon neutrality [19–21]. Many scholars have begun to discuss the effects of LS on the properties of concrete from the aspects of the hydration process and microstructure. Zhao et al. [22] used LS as a mineral admixture to replace cement, and the results showed that it can densify the microstructure of the concrete interface transition zone (ITZ), reduce internal porosity, and improve the aggressiveness of chloride ions and sulfates. Li et al. [23] also showed that the incorporation of lithium slag can increase the hydration reaction, produce hydrated calcium silicate gel (C-S-H), fill the internal pores, and improve the porosity and macrostructure of concrete. The high SO_3_ content in LS limited its high proportion to replace cement [24], and the addition of LS as a supplementary cementitious composite material would adversely affect the early strength [25], but Tan et al. [26] found that the early strength of cement slurry could be improved by wet grinding of LS. Therefore, the use of LS to replace a certain amount of cementing materials can improve the mechanical properties of concrete on the one hand, and provide a new way for the sustainable development of the lithium industry on the other hand.

Today, solid waste is being generated at an exponential rate worldwide. In China, the annual output of construction waste in 2017 was about 2 billion tons [27], and it is expected that by the end of 2026, the annual output of construction waste will reach 4.0 billion tons [28]. The study found that every 1 ton of construction waste that was comprehensively utilized can reduce carbon dioxide emissions by about 0.698 tons [29]. It has been found that shredding construction and demolition waste into RFA for reuse to replace natural fine aggregate to produce new concrete is a very effective way to dispose of construction waste [30, 31].

RFA has the defects of high water absorption [32], sharp and angular particles, and high porosity [33], which also limits its application in engineering practice. How to use RFA as a building material to produce concrete has attracted the interest of many researchers and scholars. Huang et al. [34] found that replacing natural fine aggregate with RFA can improve the early strength of concrete, but the later strength is still lower than that of natural fine aggregate concrete. Kirthika et al. [35] found that the use of RFA can negatively affect the durability performance of concrete. Therefore, to make up for the shortcomings of RFA. Some researchers have tried to reprocess RFA [36–39], use fibers [40], mineral admixtures [33], and other methods [41] to improve the mechanical properties of concrete, and have achieved good results. Gao et al. [42] found that the incorporation of mineral admixture can reduce the porosity, average pore size, and maximum pore size of recycled aggregate concrete (RAC). Barragan-Ramos et al. [43] found that the incorporation of RFA into concrete increases its conductivity, but the use of 20% fly ash can mitigate the adverse effects of RFA, thereby increasing resistance to chloride ion penetration. In this study, LS was ground into powder as a mineral admixture to replace cement and added to RFA concrete, to give full play to the advantages of the volcanic ash effect of LS and make up for the defects of RFA.

At present, many scholars have studied the single materials of LS and RFA [19, 20, 44, 45], but there are relatively few studies on the coupling of the two materials to concrete. Most of the studies focus on the basic mechanical properties of lithium slag recycled coarse aggregate concrete, and there are few studies on the stress-strain curve of lithium slag recycled fine aggregate concrete. In this study, the purpose of this study is to analyze the effects of LS content and RFA substitution rate on the axial compressive strength, peak strain, stress-strain curve shape, and elastic modulus of concrete, and to propose a stress-strain curve model of lithium slag recycled fine aggregate concrete. It is found that the incorporation of an appropriate amount of LS can make up for the mechanical defects caused by the RFA so that the axial compressive strength and elastic modulus of concrete are less different from the benchmark values, which can provide a theoretical basis and reference for the application of RFA in concrete load-bearing structures in the later stage.

## 2. Experimental program

### 2.1 Materials

**Fig 1:**
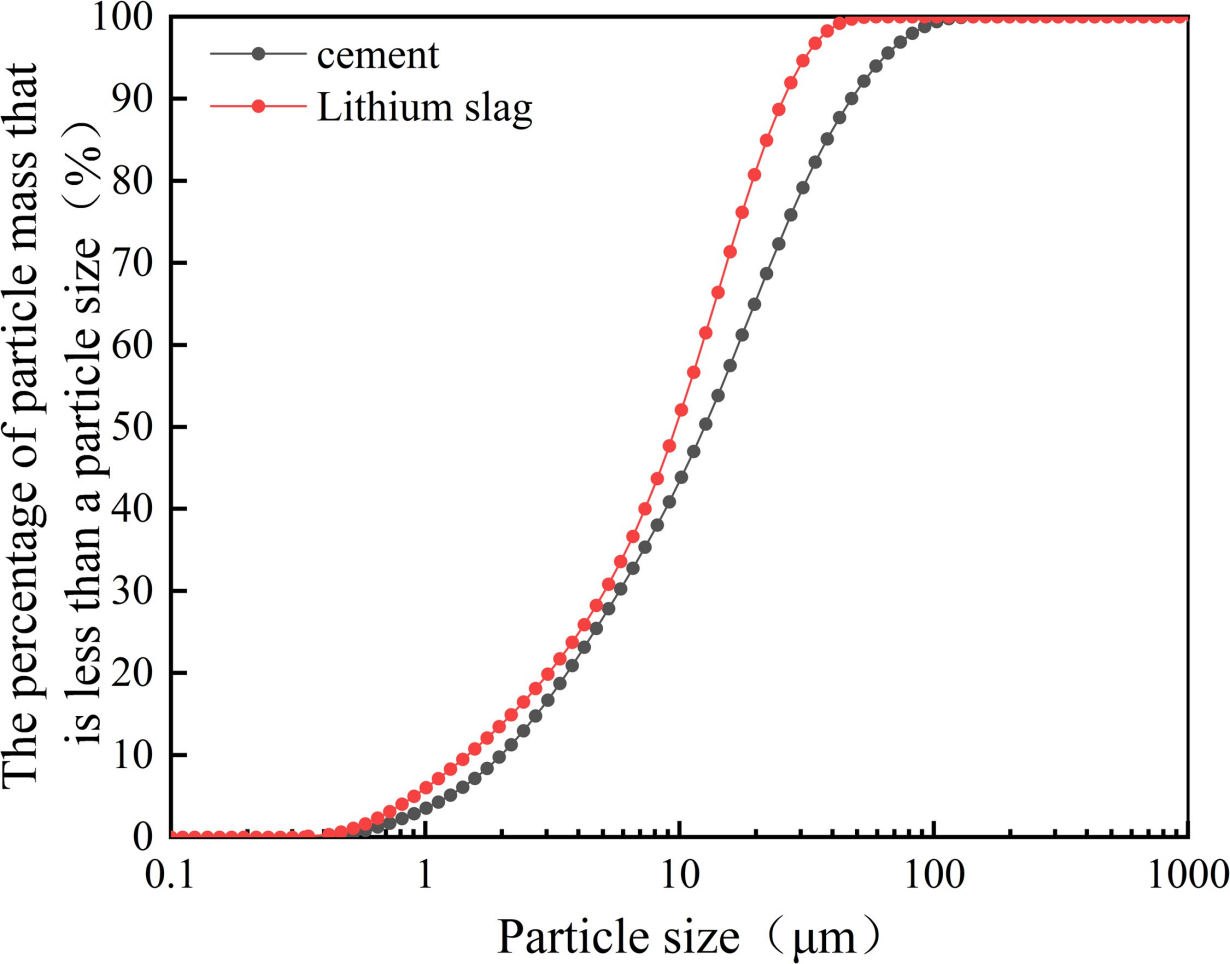
Cumulative distribution curves of cement and lithium slag particle size.

The cement used in this test is M32.5 cement, the physical properties are shown in Table 1, and the chemical composition is shown in Table 2. LS was provided by an enterprise in Wanzai County, Jiangxi Province, and the average particle size was 13.21 μm after drying and grinding by a laboratory ball mill for 1 hour, and the cumulative distribution curve of LS and cement particle size is shown in Fig 1. The specific chemical composition of LS is shown in Table 2, and the physical properties are shown in Table 3.

**Table 1:** Physical properties of ordinary Portland cement.

Test items	Fineness (%)	Stability	Clotting time (min)		Compressive strength (MPa)		Flexural strength (MPa)	
			initial setting	final set	3d	28d	3d	28d
measured value	3.2	qualified	190	350	15.8	42.6	3.2	6.8
specification value	≤10	‐‐	≥60	≤720	≥10	≥32.5	≥2.5	≥5.5

**Table 2:** Chemical composition list of cement and lithium slag (wt%).

Material	SiO_2_	CaO	Al_2_O_3_	Fe_2_O_3_	MgO	TiO_2_	SO_3_	Other
cement	61.62	21.01	4.55	3.43	1.27	0.07	2.38	5.67
LS	45.90	9.70	19.3	1.23	1.10	2.20	5.97	14.6

**Table 3:** Physical properties of lithium slag.

Performance	Specific surface area (kg/m^3^)	Apparent density (kg/m^3^)	Bulk density (kg/m^3^)	Moisture content (%)	Water absorption (%)
LS	763	1913	1065	2.16	3.67

**Fig 2:**
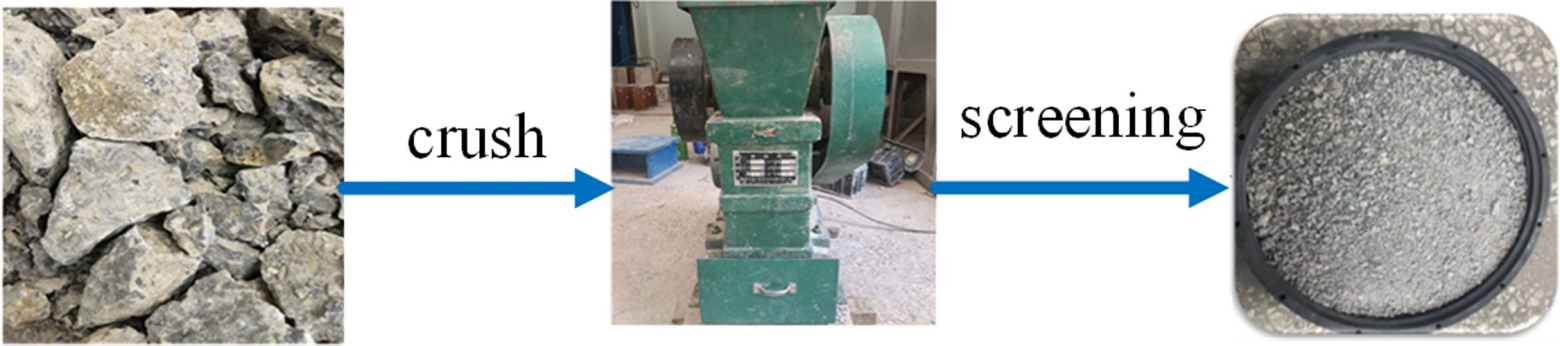
Flow chart of crushing of recycled fine aggregates.

**Fig 3:**
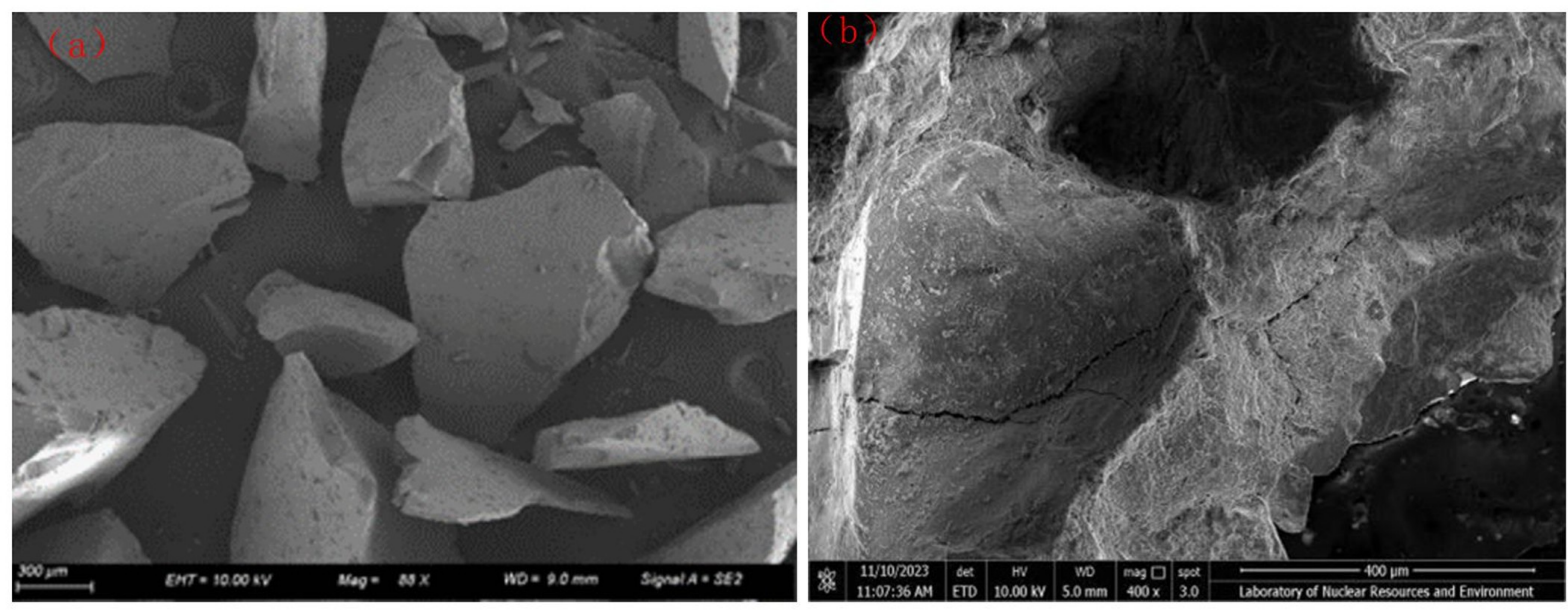
SEM micromorphology of fine aggregates. (a) RS. (b) RFA.

**Fig 4:**
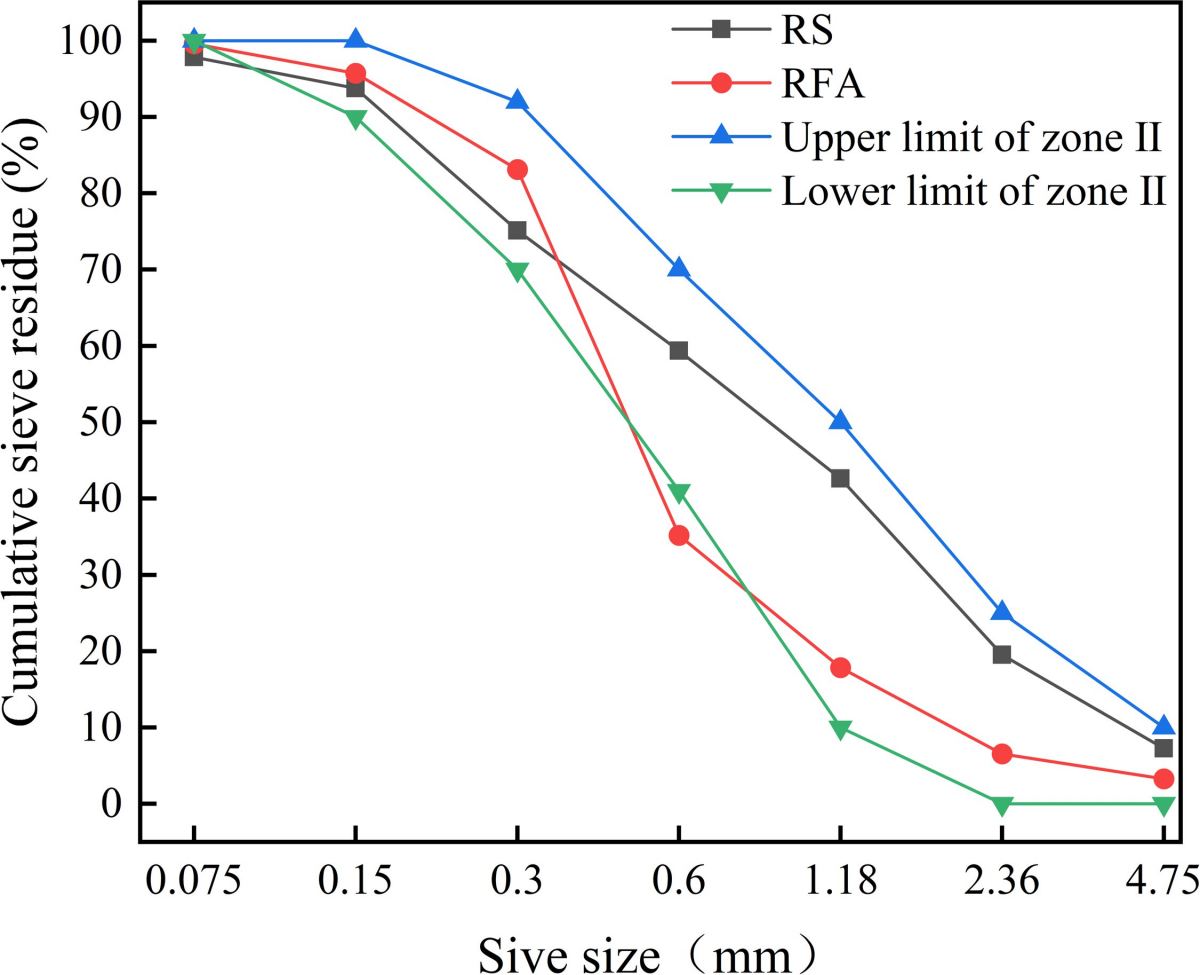
Gradation curves of different types of sand particles.

Natural river sand (RS) comes from Jiangxi Nanchang Changxin Cement Building Materials Co., Ltd., with a fineness modulus of 2.74, which belongs to the middle and in the second zone, with good gradation, and the physical properties of river sand are shown in Table 4. RFA taken from abandoned concrete blocks on broken road surfaces at East China University of Technology, automatically crushed by the crusher and then manually screened with a particle size of less than 4.75 mm, its fineness modulus is 2.18, the flow chart of RFA crushing is shown in Fig 2, in order to ensure that the surface reaches a saturated and wet state, the RFA used in the test is soaked in water for 24 hours, and its physical properties are shown in Table 4. SEM plots of RS and RFA are shown in Fig 3, the particle size sieving curve is shown in Fig 4.

**Table 4:** Physical properties of fine aggregate.

Performance	Apparent density (kg/m^3^)	Bulk Density (kg/m^3^)	Moisture content (%)	Water absorption (%)	Mud content (%)	Fineness modulus
RS	2497	1479	2.93	2.31	0.82	2.73
RFA	2361	1346	5.84	9.78	‐‐	2.18

**Fig 5:**
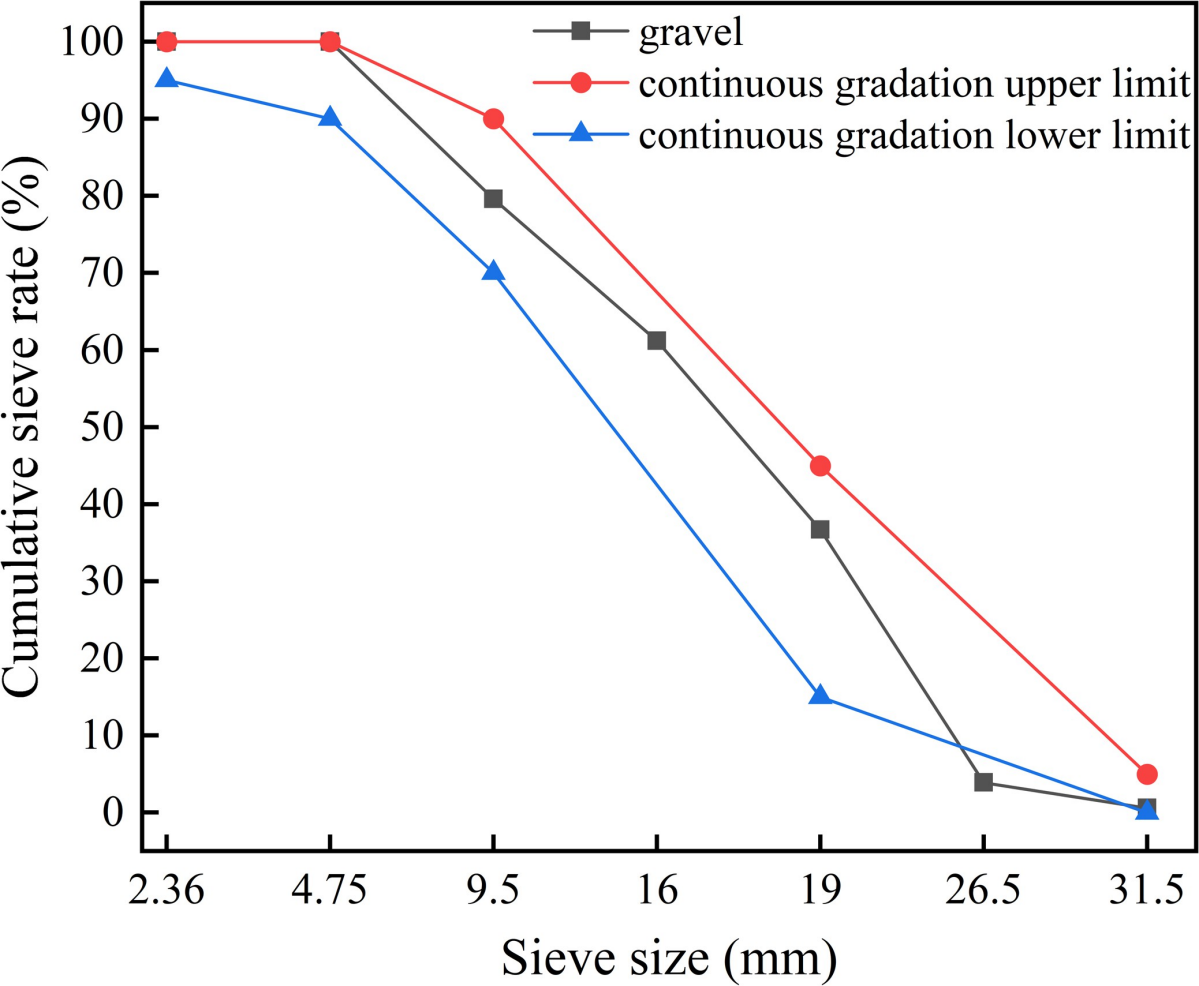
Grading curve of crushed stone.

The coarse aggregate was provided by Jiangxi Nanchang Changxin Cement Building Materials Co., Ltd. In China, the maximum particle size did not exceed 31.5mm. The physical properties of the coarse aggregate are shown in Table 5, the grading curve is shown in Fig 5.

**Table 5:** Physical properties of coarse aggregate.

Performance	Apparent density (kg/m^3^)	Bulk density (kg/m^3^)	specific gravity	Crushing index (%)	Water absorption (%)	Maximum particle size (mm)
gravel	2942	1679	1.8	7.9	1.38	31.5

The water used in this laboratory is the tap water provided by the structural laboratory, which meets the requirements of the concrete mixed water specification JGJ63-2006 through the relevant tests, and the water quality analysis results of the mixing water are shown in Table 6.

**Table 6:** Water quality analysis results of mixing water.

Test items	pH	Insoluble matter (mg/L)	Soluble (mg/L)	Cl^-^	SO_4_^2-^
measured value	7.24	1358	2193	679	1315
specification value	≥4.5	≤5000	≤10000	≤3500	≤2700

### 2.2 Mix proportions

In this paper, the influence of two factors, LS partially replacing cement mass (0, 10%, 20%, 40%) and RFA partial substitution of river sand mass (0, 10, 20%, 30%) on concrete prismatic specimens. A total of 16 groups of different mix ratios were designed, and 48 pieces of 100mm×100mm×300mm non-standard prismatic specimens were made for measuring the axial compressive strength of prism and the stress-strain relationship curve. There is a certain conversion coefficient between the axial compressive strength of standard prism specimens and non-standard prismatic specimens, with a conversion coefficient of 0.95. The design strength of the test concrete is C30, the concrete trial strength is 38.225MPa, and the mix ratio is designed by JGJ55-2011 "Ordinary Concrete Mix Design Regulations", as shown in Table 7.

**Table 7:** Concrete mix design (kg/m³).

Test piece No	Substitution rate of LS%	Replacement rate of RFA%	cement	RS	LS	RFA	water	gravel
LS0-RFA0	0	0	446	525	0	0	205	1224
LS0-RFA10	0	10	446	472.5	0	52.5	205	1224
LS0-RFA20	0	20	446	420	0	105	205	1224
LS0-RFA30	0	30	446	367.5	0	157.5	205	1224
LS10-RFA0	10	0	401.4	525	44.6	0	205	1224
LS10-RFA10	10	10	401.4	472.5	44.6	52.5	205	1224
LS10-RFA20	10	20	401.4	420	44.6	105	205	1224
LS10-RFA30	10	30	401.4	367.5	44.6	157.5	205	1224
LS20-RFA0	20	0	356.8	525	89.2	0	205	1224
LS20-RFA10	20	10	356.8	472.5	89.2	52.5	205	1224
LS20-RFA20	20	20	356.8	420	89.2	105	205	1224
LS20-RFA30	20	30	356.8	367.5	89.2	157.5	205	1224
LS40-RFA0	40	0	267.6	525	178.4	0	205	1224
LS40-RFA10	40	10	267.6	472.5	178.4	52.5	205	1224
LS40-RFA20	40	20	267.6	420	178.4	105	205	1224
LS40-RFA30	40	30	267.6	367.5	178.4	157.5	205	1224

### 2.3 Preparation of test specimens and testing methods

This test specimen was mixed was a single-horizontal shaft concrete mixer. The feeding sequence of all raw materials was gravel, RS, RFA, cement, and LS, and after 90 seconds of dry mixing, all materials were dry mixed evenly, then slowly added water to the mixer, and let the concrete mixer continue to mix for 1 minute to ensure that all the materials were mixed together, and the water was evenly mixed. The test specimen should be treated with an air pump 24 hours after pouring and forming, and then placed in the water tank for 28 days of curing, then taken out and dried naturally for testing. The test used a YAW-3000 microcomputer-controlled electro-hydraulic servo-pressured testing machine, and the maximum capacity of the pressure machine was 3000 kN. During the axial compressive strength and stress-strain test of the prism, the displacement control loading mode was adopted for control, and the loading rate was set at 0.3mm/min. During the test, the computer automatically collects the load and vertical deformation data of the concrete test block.

## 3. Results and discussion

### 3.1 Axial compressive strength

**Fig 6:**
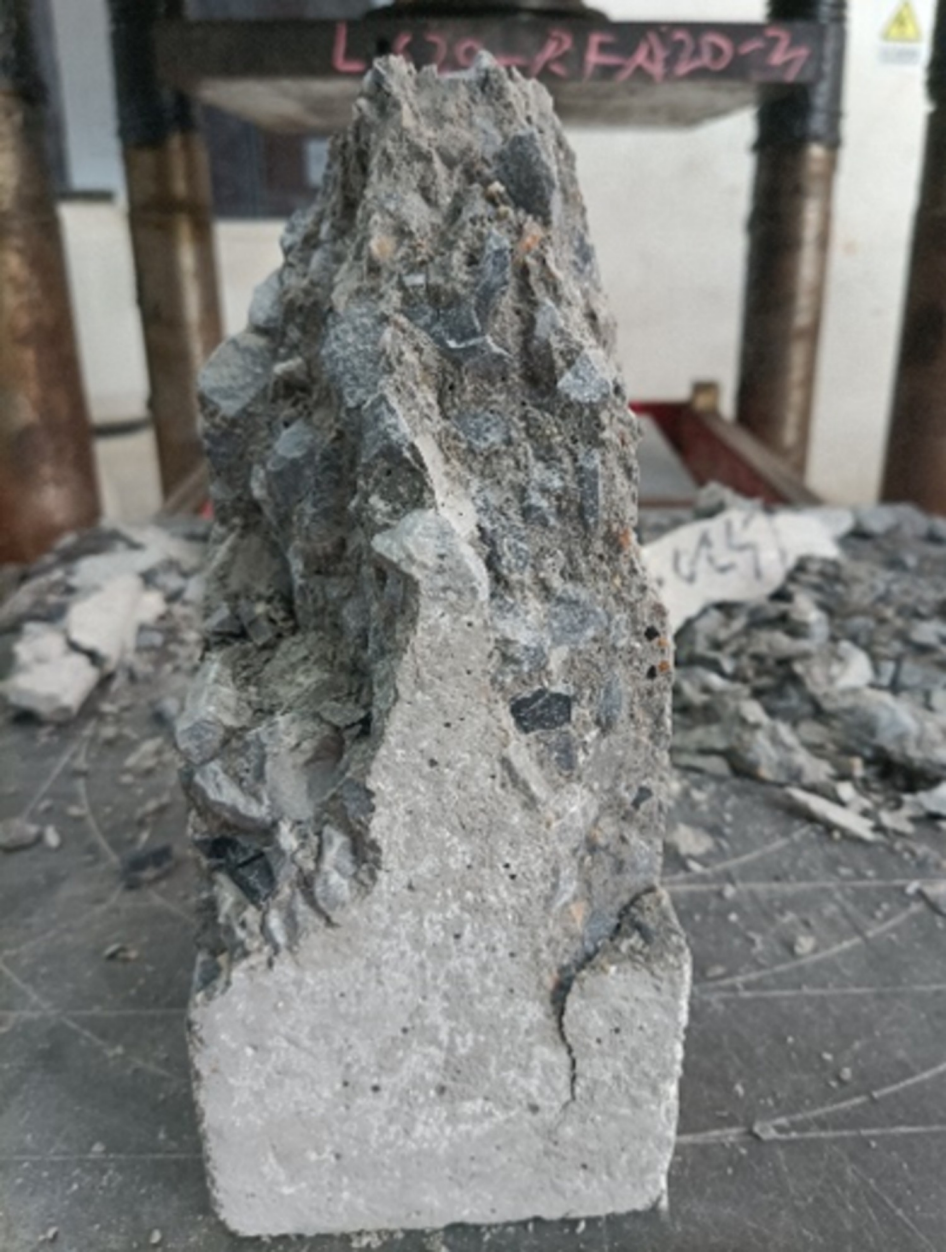
Typical failure diagram of a prism concrete under axial compression.

Fig 6 shows the typical failure mode of the axial compression of the prism of lithium slag recycled fine aggregate concrete. From the point of view of the whole failure process, when the specimen started to be loaded, there was no crack on its surface. The cracks began to develop downwards. At this time, the cracks were wider and quickly penetrated the entire test specimen. At the same time, many small cracks gradually appeared around the surface of the test specimen, followed by a violent sound, and the load value dropped rapidly. The test specimen failure occurred due to loss of bearing capacity and eventually became a cone-shaped failure, which was manifested as an obvious brittle failure. The axial compressive failure process of the lithium slag recycled fine aggregate concrete group and the benchmark concrete group was very similar. But with the increase of the replacement rate of RFA, the failure process was slightly shorter than that of no RFA, and during the failure process, the debris produced by the specimen increased. From the perspective of the internal structure of concrete failure, when the LS content is less than 20%, the failure surface is basically a fracture between the coarse and fine aggregates. When the LS content exceeds 20%, the failure surface is basically the fracture phenomenon caused by the insufficient adhesion between the coarse and fine aggregates and the cementitious material.

**Fig 7:**
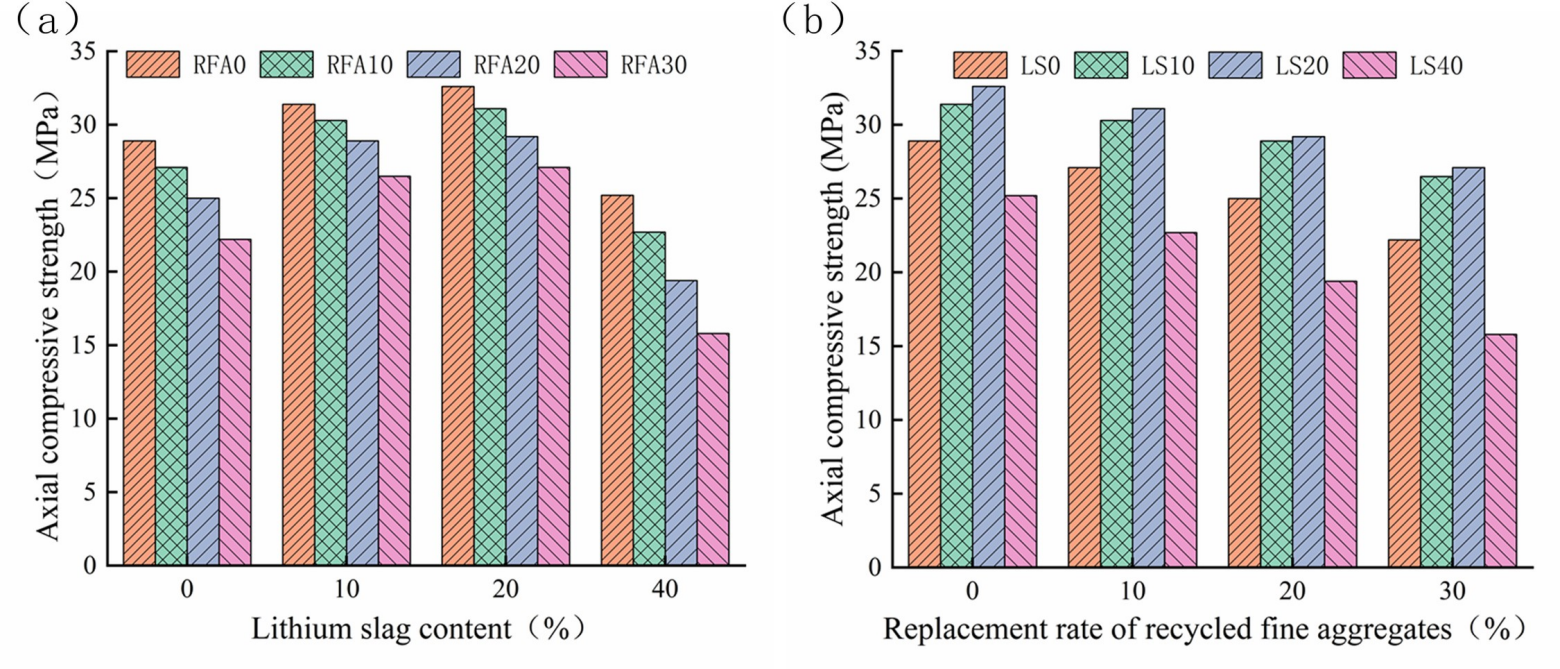
Axial compressive strength of lithium slag recycled fine aggregate concrete. (a) LS. (b) RFA.

It can be seen from Fig 7 that with the increase of LS content, the axial compressive strength of lithium slag recycled fine aggregate concrete first increases and then decreases, and its peak value is when the LS content is 20%, This is similar to the results of Qin et al [46]. The axial compressive strength of the LS content is even lower than that of the control group’s concrete axial compressive strength. The reason can be attributed to the fact that the incorporation of an appropriate amount of LS can refine the cement pores, while the excess LS cannot produce enough hydrated calcium silicate gel to fill the concrete pores, resulting in the enlargement of concrete pores [11, 13, 20]. When only the influence of the substitution rate of RFA on the axial compressive strength of concrete is considered, with the increase of the substitution rate of RFA, the axial compressive strength of RFA concrete shows a downward trend, and the more the dosage, the more obvious the downward trend [47, 48], because the RFA will produce more microcracks when it is crushed, and its composition contains fine powder, mud and harmful substances, etc., which hinder the cementation of cement and aggregate, and cannot effectively fill the gap between the coarse aggregate, resulting in the reduction of concrete compactness and the increase of porosity [49, 50]. When the two materials act together in the concrete, the appropriate amount of LS will make up for the defects caused by the RFA, such as the axial compressive strength of LS10-RFA10, LS20-RFA10 and LS20-RFA20 is higher than that of the reference group, because the chemical composition of LS contains a large amount of SiO_2_, Al_2_O_3_ and CaO, etc. These chemicals can have a secondary hydration reaction with the hydration product Ca(OH)_2_ in cement. A hydrated calcium silicate gel (C-S-H) with a certain strength is generated, which can be filled in the microcracks produced by the crushing of concrete pores and RFA, resulting in a decrease in porosity, and its axial compressive strength is higher than that of the non-mixed LS group.

### 3.2 Elastic modulus

**Fig 8:**
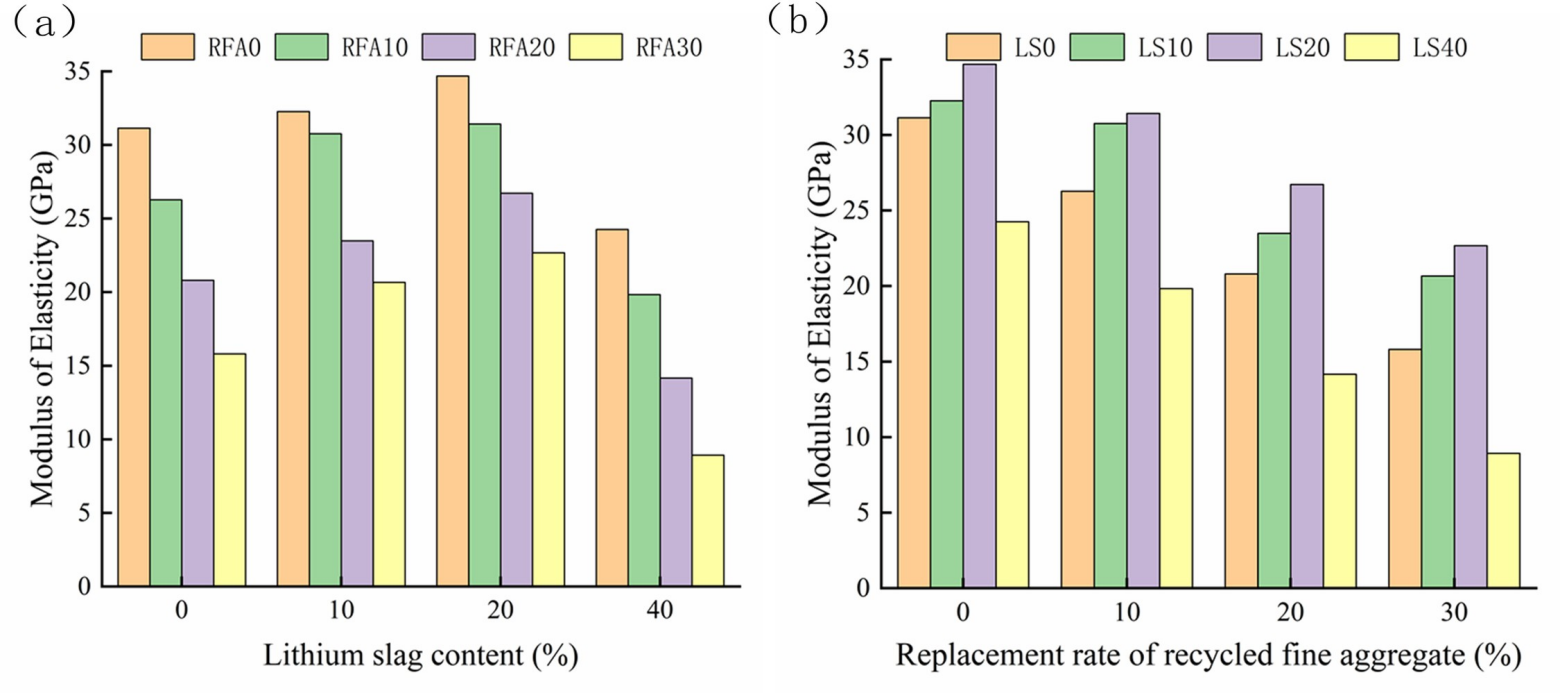
Elastic modulus value of lithium slag recycled fine aggregate concrete. (a) LS. (b) RFA.

The elastic modulus E_C_ of concrete is defined as the ratio of compressive strain to stress under unidirectional compression, and the secant modulus from the initial point to the 0.4f_cp_ point in the rising section of the stress-strain curve is generally taken as the E_C_ value. Fig 8 shows the result value of the elastic modulus of lithium slag recycled fine aggregate concrete. Compared with the axial compressive strength results of the prism, it is found that the elastic modulus value is very similar to the axial compressive strength value of the prism. With the increase of LS content, the elastic modulus of concrete increases first and then decreases, and when the LS content is 20%, its elastic modulus reaches the optimum [46, 51]. With the increase of the RFA substitution rate, its elastic modulus decreases with the increase of the RFA substitution rate [52–54]. Compared with RFA0, RFA10, RFA20 and RFA30 decreased by 15.61%, 33.17% and 49.23% compared with RFA0 when the LS content was 0%. When the LS content was 10%, it decreased by 4.68%, 27.21% and 35.95%. When the LS content was 20%, it decreased by 9.37%, 22.92%, and 34.60%. When the LS content was 40%, it decreased by 18.22%, 41.63%, and 63.19%. The cause analysis is consistent with the analysis of the cause of the axial compressive strength of the lithium slag recycled fine aggregate concrete prism, and will not elaborate too much here.

### 3.3 Stress-strain relationship

**Fig 9:**
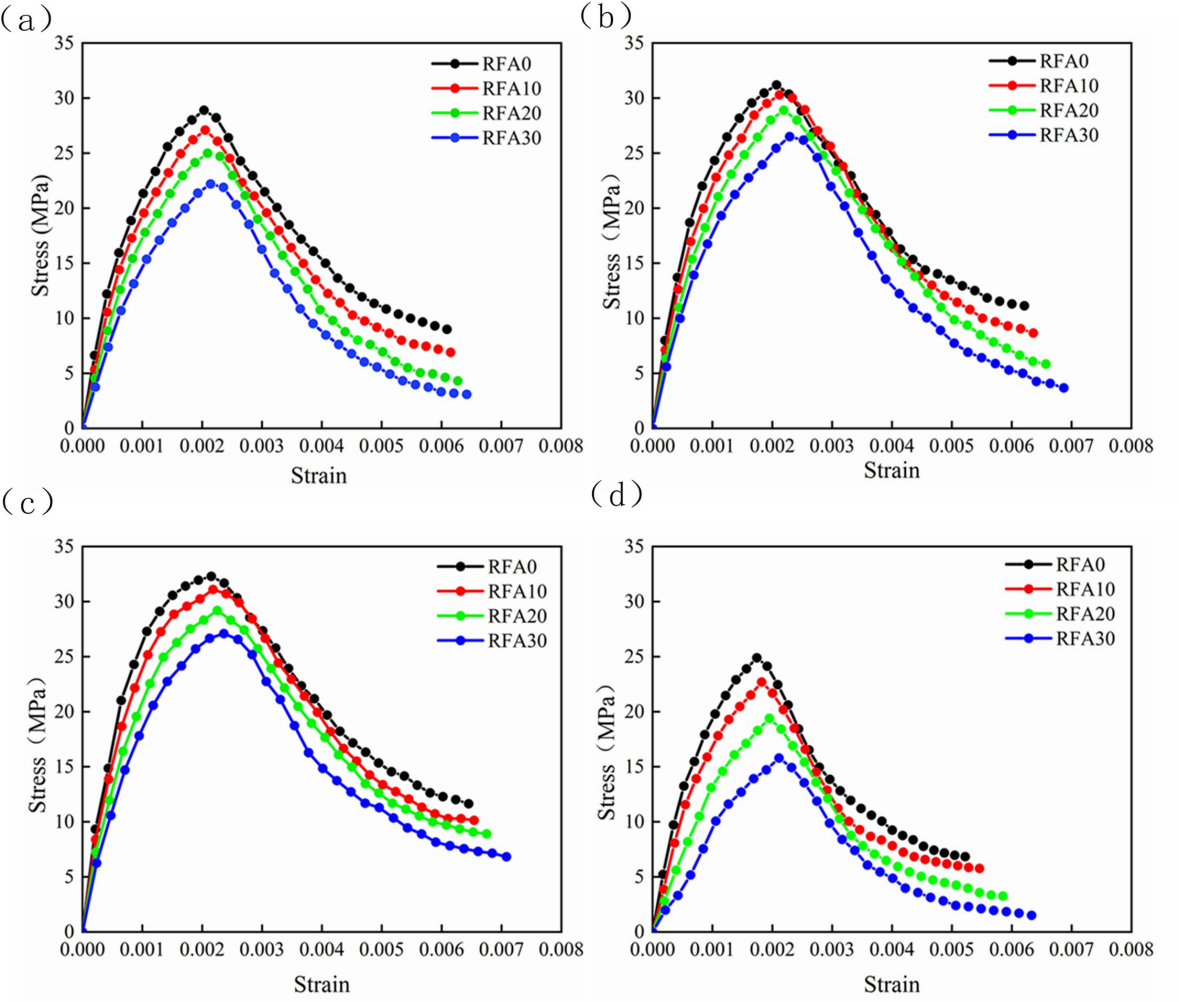
Stress-strain curves of concrete with different LS contents. (a) Sample with 0% LS content. (b) Sample with 10% LS content. (c) Sample with 20% LS content. (d) Sample with 40% LS content.

The stress-strain relationship curve between LS content and concrete is shown in Fig 9. The stress-strain curves of concrete show similar rules regardless of the substitution rate of RFA, which are composed of rising and descending sections. The analysis of Fig 9 shows that the curve of the ascending section shows great differences when different RFA is applied. With the increase of the substitution rate of RFA, the curve slope will be more gentle, which also indicates that its elastic modulus is decreasing. In the descending section of the stress-strain curve, there is also a certain difference when the RFA is regenerated with different substitution rates, and the curve changes in the descending section are steep at first and then gradually tends to be flat when the substitution rate of the RFA increases. This is because the RFA itself has many cracks and high porosity defects, the higher the substitution rate, the more it shows the characteristics of crushing, which makes the curve rise section perform steeper, with the increase of the load, some of the RFA is completely crushed, filled in the concrete void, so that the curve in the descending section is more gentle.

**Fig 10:**
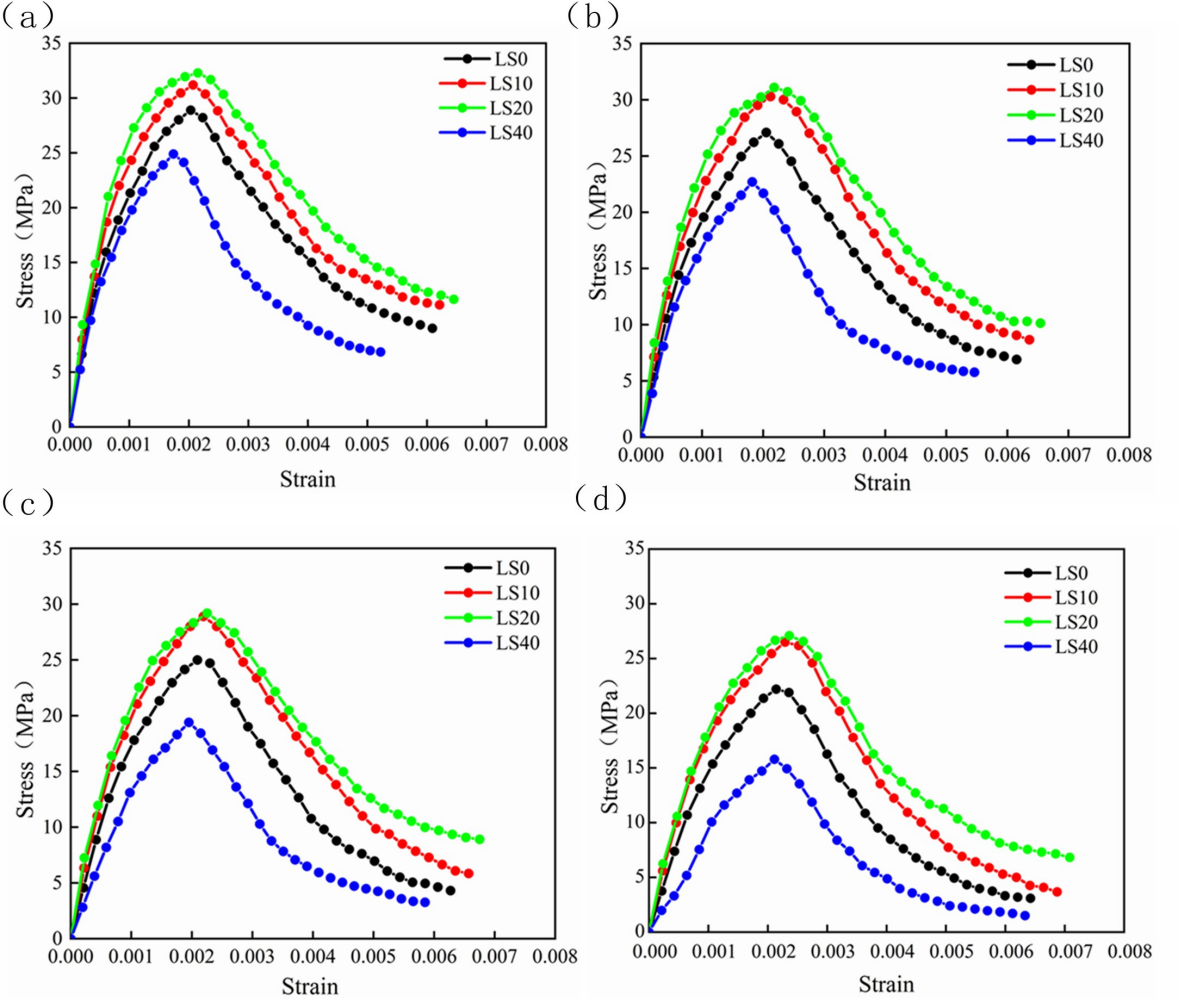
Stress-strain curve of concrete with different replacement rates of RFA. (a) Sample with a replacement rate of 0% for RFA. (b) Sample with a replacement rate of 10% for RFA. (c) Sample with a replacement rate of 20% for RFA. (d) Sample with a replacement rate of 30% for RFA.

The relationship between the substitution rate of RFA and the stress-strain curve is shown in Fig 10. It can be seen from the observation that when the LS content was within the range of 20%, the slope of the rising curve became steeper with the increase of LS content. The gentler the peak stress was, the higher the peak stress would be. When the amount of LS was further increased, the slope of the curve of the rising section began to became flat, and the peak stress would show a downward trend; in the stress-strain falling section, the curve was similar to that of the rising section. This also indirectly showed that the amount of LS within a certain range could react with cement to produce C-S-H [20, 55], which could be filled in the interior of the concrete to make up for the natural defects such as micro-cracks in the RFA, thereby improving the ductility of concrete and delaying concrete failure.

### 3.4 Peak strain

**Fig 11:**
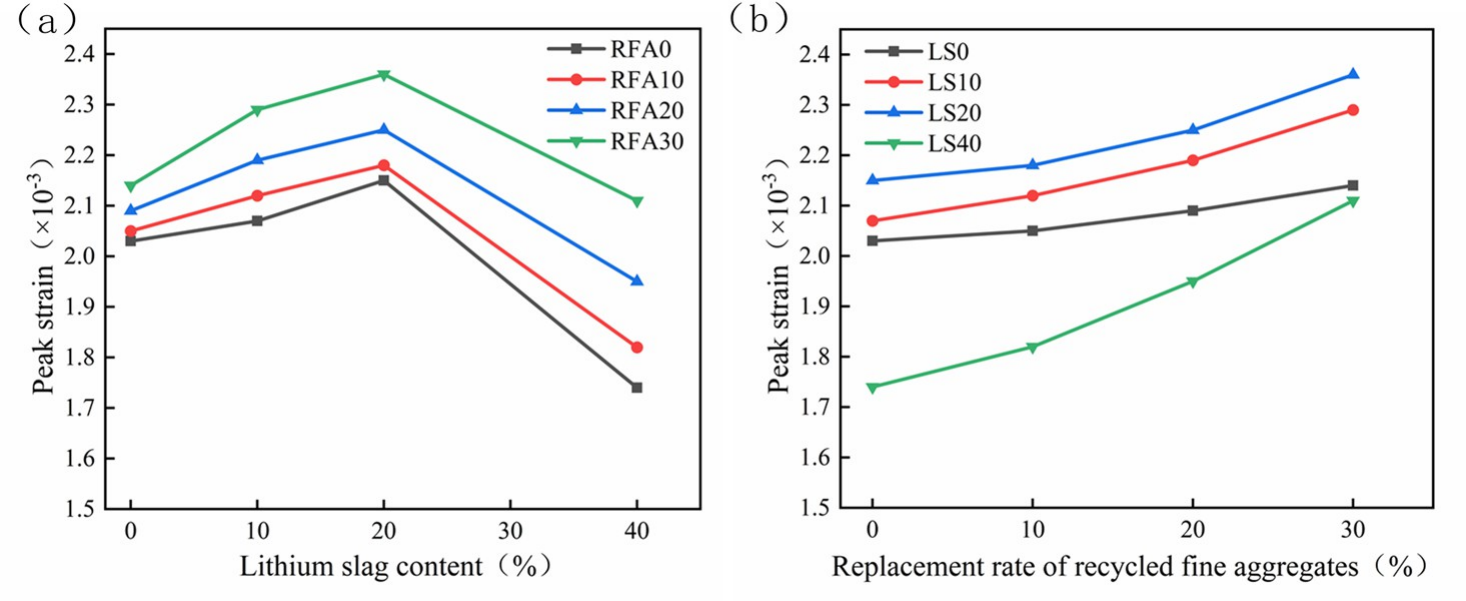
Peak strain value of lithium slag recycled fine aggregate concrete.

The influence of LS content and RFA substitution rate on the peak strain of concrete is shown in Fig 11. Compared with the preference concrete, whether LS or RFA was added, the peak strain increased continuously. The peak strains of RFA10, RFA20, and RFA30 increased by 0.99%, 2.96%, and 5.42%. When the LS content was 0%, respectively; when the LS content was 10%, it increased by 4.43%, 7.88%, and 12.81%, respectively. When the LS was 20%, it increased by 7.39%, 10.84%, and 16.26% respectively; when the LS was 40%, it increased by 4.60%, 12.07%, and 21.26%, respectively. This may be because as the RFA substitution rate increased, the cement base adhered to the aggregate surface increased, so that the actual gel content of the RFA concrete increased after solidification, so its peak strain increased [52, 56]. When only considering the effect of LS content on the peak strain of concrete, LS10 and LS20 increased by 1.97% and 5.91% compared with the reference group [46, 51]; while LS40 decreased by 14.29% compared with the reference group. The reason can be attributed to the fact that an appropriate amount of LS can effectively fill the voids of concrete, improve its compactness, weaken its brittleness, increase its ductility, and thus increase its peak strain. While an excessive amount of LS will cause porosity due to insufficient supply of hydration products. So, it results in increased brittleness and a decrease in peak strain.

### 3.5 Prediction of constitutive equation

This experiment was fitted using the constitutive model formula of concrete under uniaxial compression proposed by Guo [57], which is:

$$y=\left\{\begin{array}{c} Ax+(3-2A)x^{2}+(A-2)x^{3},0\leq x<1\\ \frac{x}{B{\left(x-1\right)}^{2}+x},x\geq 1 \end{array}\right.$$ (1)

In Eq (1) *x* = *ε* / *ε*_0_、*y* = *σ* / *f*_*c*_, A and B represent the parameters of the rising and falling curves, respectively. Parameter A reflects the initial elastic modulus of lithium slag recycled fine aggregate concrete, while parameter B is related to the area of the falling part of the stress-strain curve. The larger the value of A, the smaller the value of B, indicating that the concrete breaks slowly and the material ductility is better. The smaller the value of A, the larger the value of B, indicating that the concrete material is more brittle and has less plastic deformation.

**Fig 12:**
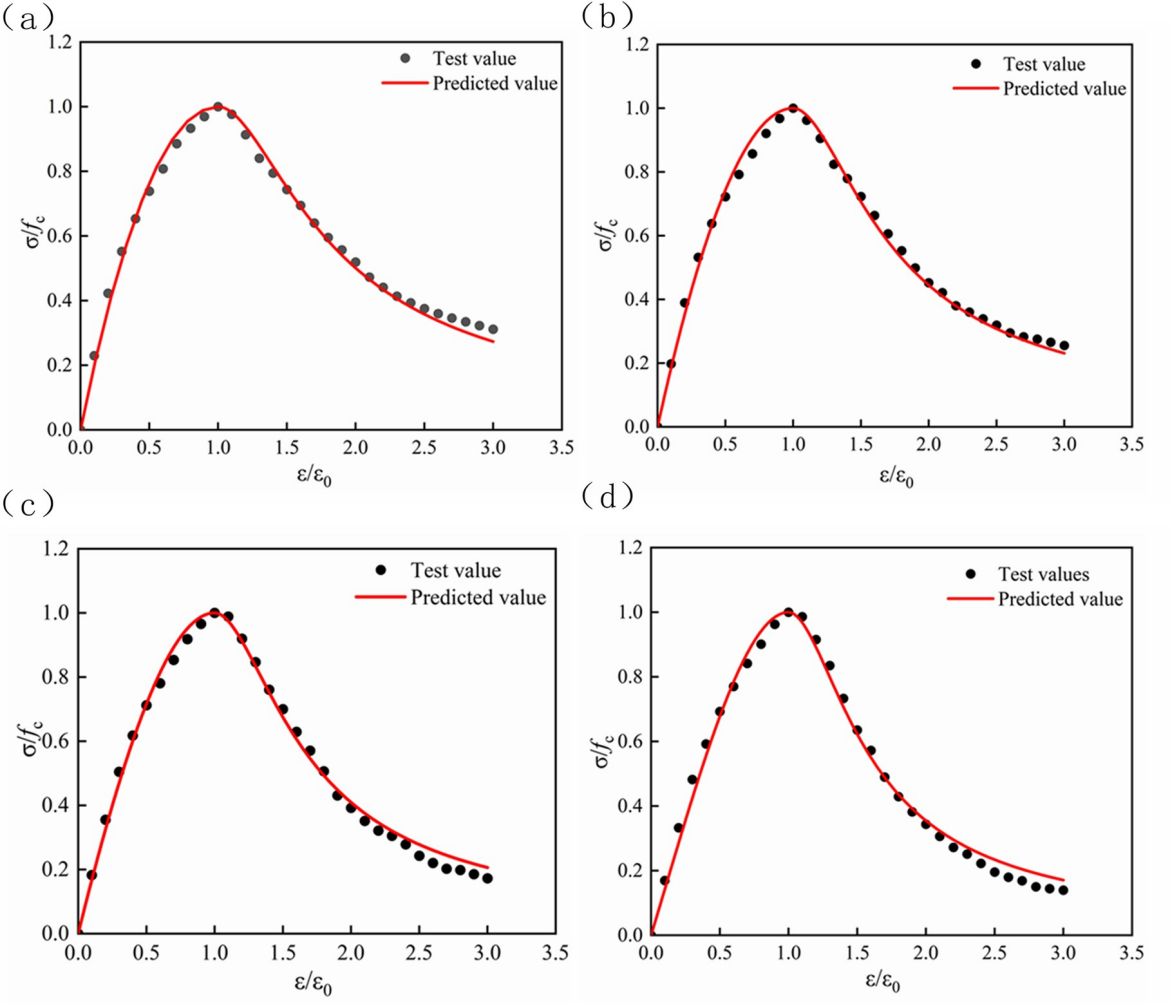
Comparison of LS0 normalized stress-strain curve. (a) RFA = 0. (b) RFA = 10. (c) RFA = 20. (d) RFA = 30.

**Fig 13:**
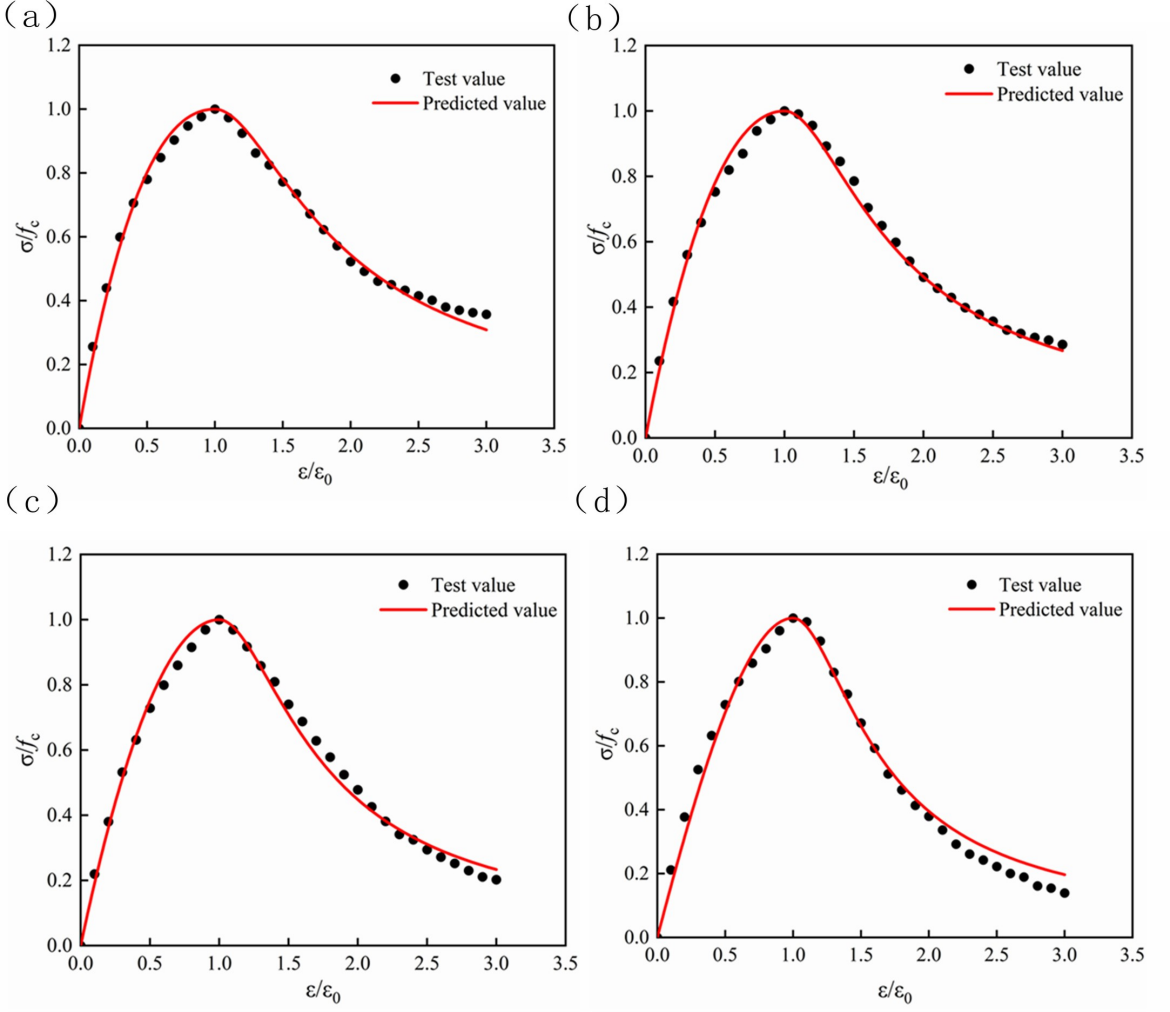
Comparison of LS10 normalized stress-strain curve. (a) RFA = 0. (b) RFA = 10. (c) RFA = 20. (d) RFA = 30.

**Fig 14:**
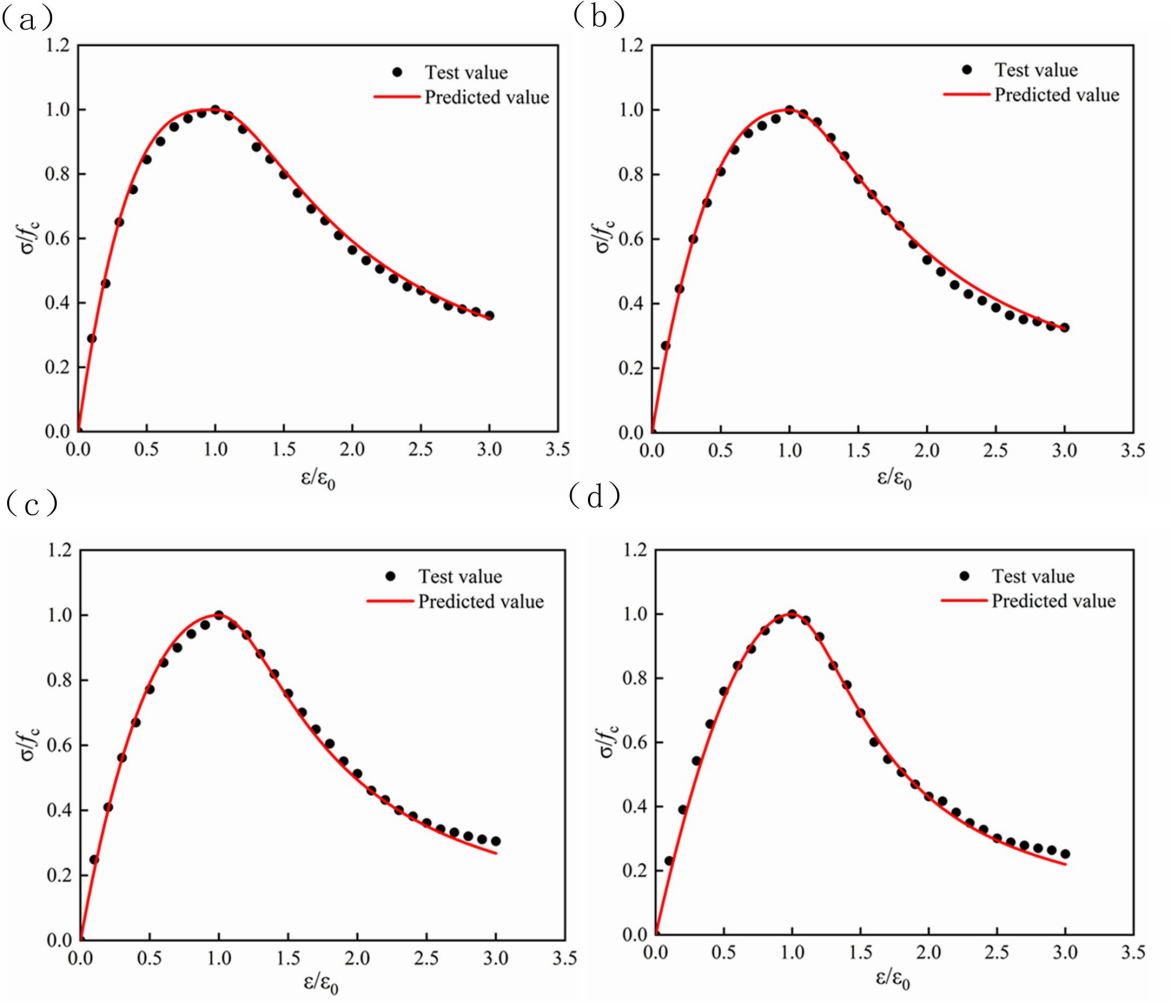
Comparison of LS20 normalized stress-strain curve. (a) RFA = 0. (b) RFA = 10. (c) RFA = 20. (d) RFA = 30.

**Fig 15:**
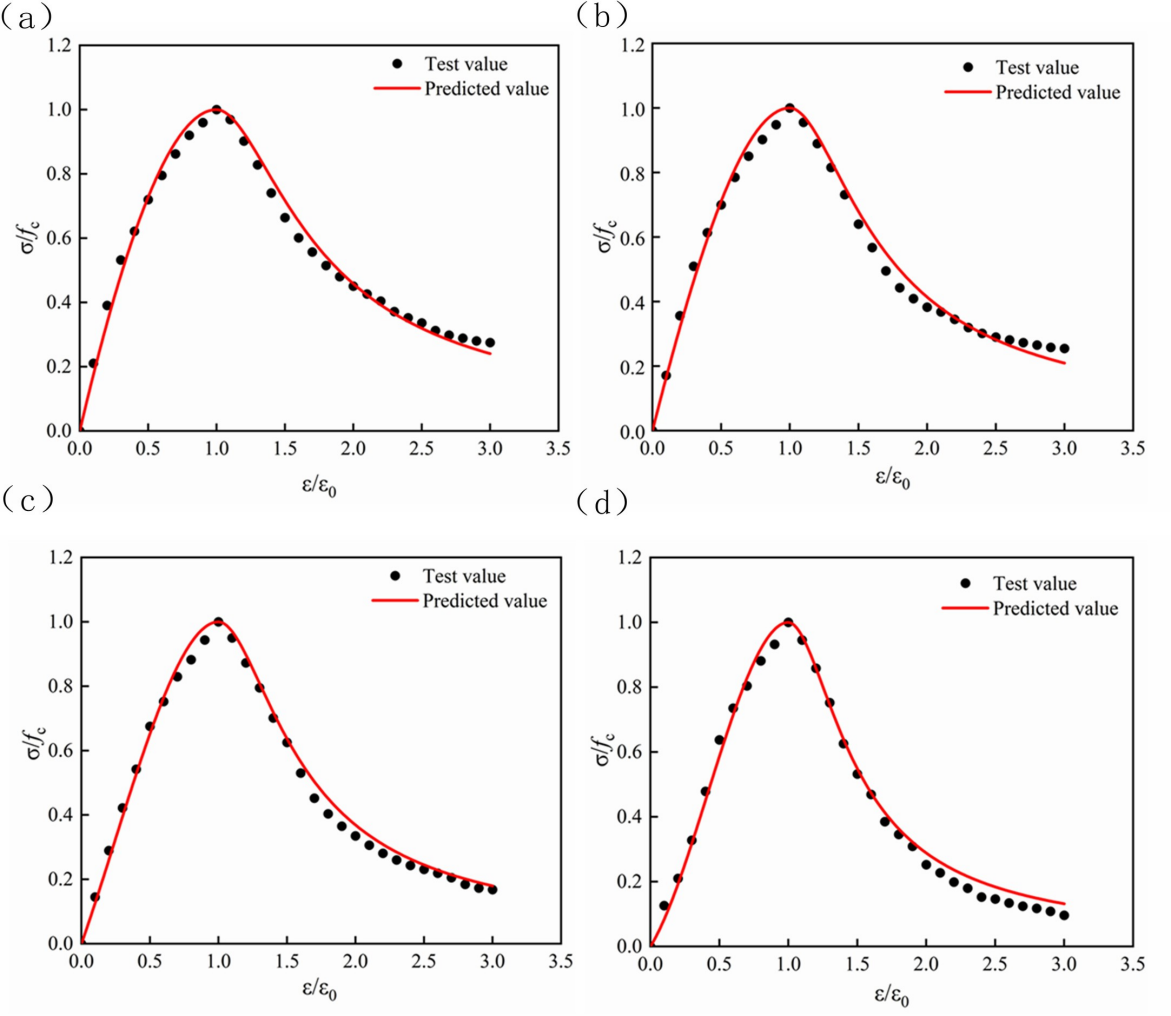
Comparison of LS40 normalized stress-strain curve. (a) RFA = 0. (b) RFA = 10. (c) RFA = 20. (d) RFA = 30.

The stress-strain relationship of lithium slag recycled fine aggregate concrete was obtained through experiments, and the least square method was used to fit the rising and falling sections of the stress-strain curve with different LS content and RFA (Figs 12–15). The values of parameters A and B and the correlation coefficient R^2^ under the condition of aggregate substitution rate are shown in Table 8. It can be seen from Table 8 that the correlation coefficient R^2^ of the obtained parameters A and B values were all above 0.95, which could be used for subsequent projects. The application provided reference value.

**Table 8:** Stress-strain fitting results.

Specimen number	A	R^2^	B	R^2^
LS0-RFA0	2.11675	0.99172	1.99269	0.98726
LS0-RFA10	1.94510	0.98934	2.49571	0.99251
LS0-RFA20	1.72704	0.99473	2.68758	0.98464
LS0-RFA30	1.41727	0.98331	3.63730	0.97883
LS10-RFA0	2.41518	0.99583	1.67912	0.98536
LS10-RFA10	2.24130	0.98936	2.05762	0.99031
LS10-RFA20	2.01431	0.99259	2.46261	0.97965
LS10-RFA30	1.63550	0.97758	3.06267	0.96862
LS20-RFA0	2.99250	0.99671	1.30819	0.98652
LS20-RFA10	2.61451	0.99563	1.57322	0.98011
LS20-RFA20	2.32890	0.99737	2.04755	0.99062
LS20-RFA30	1.89130	0.99813	2.66082	0.98822
LS40-RFA0	1.82192	0.99095	2.36721	0.97819
LS40-RFA10	1.68610	0.98897	2.83195	0.96157
LS40-RFA20	1.28833	0.99383	3.41922	0.98271
LS40-RFA30	0.69173	0.98576	4.93751	0.95767

The parameters A、B, LS content, and the replacement rate of RFA were fitted, and the relationship was obtained as follows:

$$A=-19.49r1^{2}-4.96r2^{2}-4.11r1r2+7.50r1-0.87r2+2.05$$ (2)

$$B=25.97r1^{2}+12.94r2^{2}+8.48r1r2-10.16r1+0.26r2+2.28$$ (3)

Formulas (2) and (3) *r*_1_ represent the amount of LS and *r*_2_ the replacement rate of RFA.

**Fig 16:**
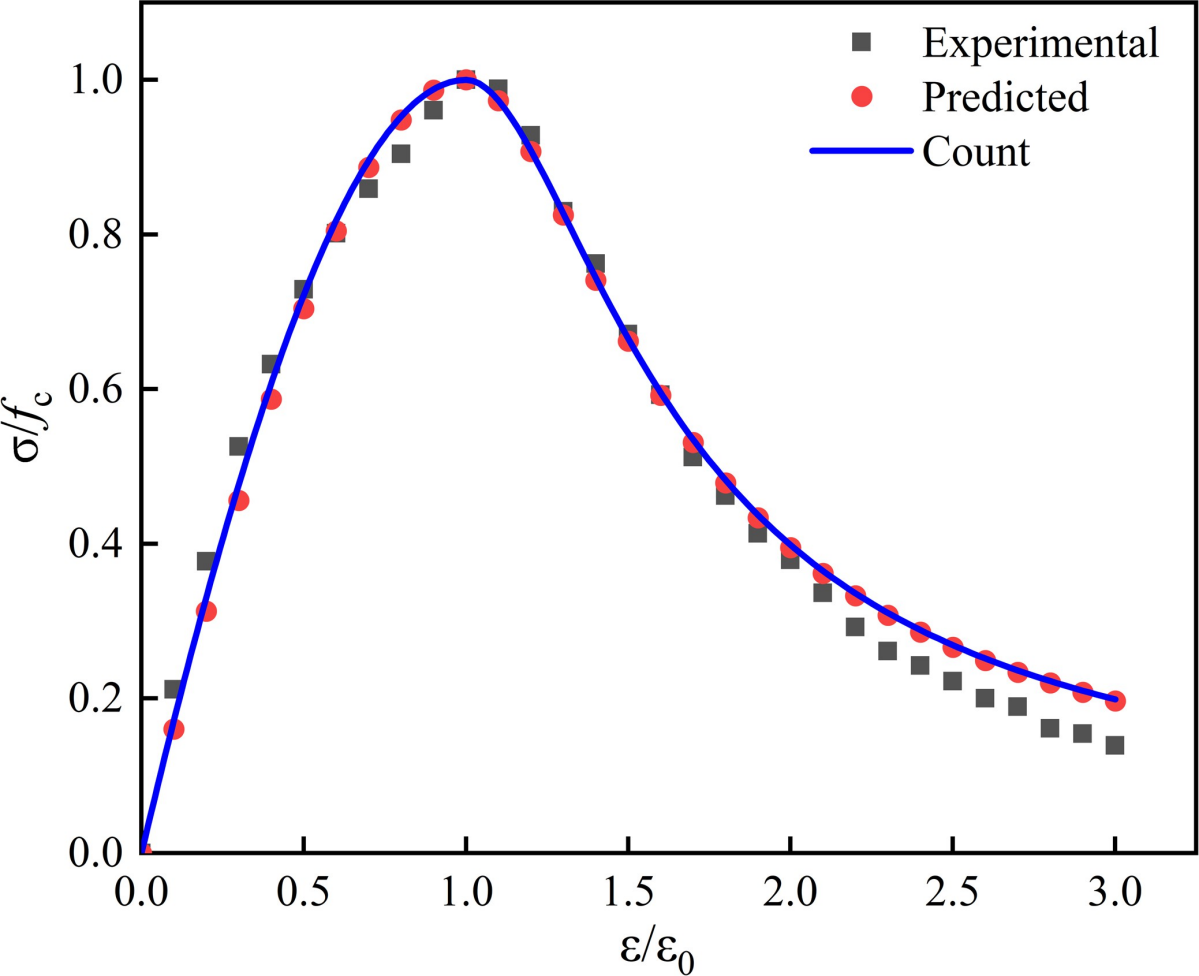
Comparison of three stress-strain curves.

Use formula (2) and formula (3) to calculate A and B values, and substitute them into formula (1) to get the calculated stress-strain full curve, the measured full curve, and the full curve predicted by linear regression (taking LS10RFA30 specimen as an example). As shown in Fig 16, it can be seen that from the measured stress-strain curve, the predicted full curve had a linear regression, and the calculated stress-strain full curve had a high degree of agreement.

## 4. Conclusions

In this study, the influence of LS replacing 0%, 10%, 20%, and 40% cement material and RFA replacing 0%, 10%, 20%, and 30% RS in concrete on axial compressive strength and stress-strain curve was studied. Our conclusions were summarized as follows:

1. Similar to the failure form of the concrete in the reference group, the prism specimens of lithium slag recycled fine aggregate concrete also showed a cone-shaped failure form in the end, and the whole process showed obvious brittle failure.
2. With the increase of LS content, the axial compressive strength, elastic modulus, and peak strain of concrete showed a trend of first increasing and then decreasing. When the LS content was 20%, all specimens reached their optimal state. With the increase of the replacement rate of RFA, the axial compressive strength and elastic modulus of concrete showed a decreasing trend, while the peak strain showed the opposite trend, which increased with the increase of the replacement rate of RFA.
3. An appropriate amount of LS is incorporated into the concrete to make up for the mechanical defects caused by the RFA so that the axial compressive strength and elastic modulus values are less different than those of the reference group.
4. The stress-strain curve equation of lithium slag recycled fine aggregate concrete (i.e., (Eq 1)) is proposed, which has a high degree of agreement compared with the test results and can provide a reference for practical engineering applications.
5. Based on the A and B values in the proposed Eq (1), the relationship between the LS content and the substitution rate of RFA is fitted to obtain Eqs (2 and (3), which are compared with the test results and Eq (1), and the error is small.

## Supporting information

S1 Dataset (XLS)
